# Molecular Links Between Angiogenesis and Neuroendocrine Phenotypes in Prostate Cancer Progression

**DOI:** 10.3389/fonc.2019.01491

**Published:** 2020-01-21

**Authors:** Zheng Wang, Yicheng Zhao, Zhiqiang An, Wenliang Li

**Affiliations:** ^1^Texas Therapeutics Institute, Brown Foundation Institute of Molecular Medicine, University of Texas Health Science Center at Houston (UTHealth), Houston, TX, United States; ^2^MD Anderson Cancer Center UTHealth Graduate School of Biomedical Sciences (GSBS), Houston, TX, United States

**Keywords:** new therapeutics, molecular mechanisms, angiogenesis, neuroendocrine prostate cancer, neuroendocrine phenotype

## Abstract

As a common therapy for prostate cancer, androgen deprivation therapy (ADT) is effective for the majority of patients. However, prolonged ADT promotes drug resistance and progression to an aggressive variant with reduced androgen receptor signaling, so called neuroendocrine prostate cancer (NEPC). Until present, NEPC is still poorly understood, and lethal with no effective treatments. Elevated expression of neuroendocrine related markers and increased angiogenesis are two prominent phenotypes of NEPC, and both of them are positively associated with cancers progression. However, direct molecular links between the two phenotypes in NEPC and their mechanisms remain largely unclear. Their elucidation should substantially expand our knowledge in NEPC. This knowledge, in turn, would facilitate the development of effective NEPC treatments. We recently showed that a single critical pathway regulates both ADT-enhanced angiogenesis and elevated expression of neuroendocrine markers. This pathway consists of CREB1, EZH2, and TSP1. Here, we seek new insights to identify molecules common to pathways promoting angiogenesis and neuroendocrine phenotypes in prostate cancer. To this end, our focus is to summarize the literature on proteins reported to regulate both neuroendocrine marker expression and angiogenesis as potential molecular links. These proteins, often described in separate biological contexts or diseases, include AURKA and AURKB, CHGA, CREB1, EZH2, FOXA2, GRK3, HIF1, IL-6, MYCN, ONECUT2, p53, RET, and RB1. We also present the current efforts in prostate cancer or other diseases to target some of these proteins, which warrants testing for NEPC, given the urgent unmet need in treating this aggressive variant of prostate cancer.

## Introduction

In the United States, prostate cancer is responsible for the second most cancer death in men, behind lung cancer. It is estimated that about 31,620 deaths in 2019 in USA are caused by prostate cancer (www.cancer.org). Androgen deprivation therapies (ADT) that target the androgen receptor (AR) is the main treatment for prostate cancer (1–4). ADT is effective initially. However, the majority of tumors invariably relapse and progress, becoming castration resistant prostate cancer (CRPC) (1–4). Frequently associated with ADT resistance is the emergence of neuroendocrine prostate cancers (NEPC) that have a poor prognosis with no effective treatment (5–8). With the common use of new generation potent ADT into clinic, the incidence of NEPC is rising (6, 9–12).

NEPC are highly vascularized (13, 14). Increased angiogenesis and expression of NE markers are two prominent phenotypes of NEPC (13–16) and are expected to be molecularly linked. However, direct molecular connections between these two phenotypes in NEPC remain largely unclear. The main purpose of this review is to summarize the reported and potential connections between the regulation of increased angiogenesis and expression of NE markers. Further, we analyze the implications of these connections for prostate cancer. Our goal is to identify key regulators of both characteristics as potential targets for NEPC, with the hope of hitting two birds with one stone to achieve better therapeutic efficacy and fewer side effects.

## Neuroendocrine Phenotype in Prostate Cancer

Approximately 20% of lethal CRPCs have a neuroendocrine (NE) phenotype, and thus are called NEPC or CRPC-NE (5, 17–19). NEPCs often lose AR signaling, become resistant to ADT, and express NE markers, such as neuron-specific enolase 2 (ENO2), synaptophysin (SYP), chromogranin A and B (CHGA and CHGB) (5–8). Features of NEPC include elevated angiogenesis, high proliferative rates, and metastatic propensity (20). Unfortunately, there is no effective therapy against NEPCs. They respond only transiently to chemotherapy (17, 20–24).

Clinical data, including genomic analyses of metastatic tumors, and preclinical studies suggest an evolution of CRPC-NE from a prostate adenocarcinoma precursor (25–27). Researchers are beginning to unfold the signaling events involved in NEPC development (6, 17, 24). General knowledge of NEPC has been elegantly reviewed (19, 20, 24, 28–32). A number of proteins have been reported to contribute to NEPC progression. These proteins include Aurora kinase A and B, BRN2, CREB1, DEK, EZH2, FOXA2, GRK3, HIF1, IL-6, MYCN, ONECUT2, PEG10, p53, REST, RB1, SRRM4, SOX2 et al. (33–44).

## Angiogenesis in Prostate Cancer

As a basic physiological process, angiogenesis usually occurs in embryonic development, tissue repair and fertility to form new blood vessels resulted from the extension of pre-existing vasculature. In addition, angiogenesis is also accompanied by chronic inflammation, tumor growth and metastasis (19). Actually, angiogenesis is a dynamic process involves interaction between endothelial cells and their extracellular environment. There are two main types of angiogenesis *in vivo*, including sprouting angiogenesis (sprouting of vascular endothelia from pre-existing capillary endothelia into the surrounding tissues) and non-sprouting angiogenesis (division of pre-existing capillaries by tissue pillars into new daughter vessels) (19, 45–47). The formation of new blood vessels depends on a balanced process that are regulated by many factors (48). Angiogenic activators include angiopoietins, CCL2, EGFL6, endothelins, FGF, HIF1, IGF1, MMPs, PDGF, TGF, VEGF, and et al. (48–56). On the other hand, angiostatin, endostatin, TSP1, and PAI2 are among the endogenous inhibitors of angiogenesis (57–60).

Angiogenesis is involved in prostate cancer survival, progression, and metastasis (61). Its importance in prostate cancer has been established (62, 63). Higher microvessel density is associated with worse prognosis in prostate cancer (64, 65). VEGF as well as some neurosecretory peptides, e.g., serotonin, bombesin, and gastrin, have been shown to boost angiogenesis in NEPC (15). We recently reported that ADT repression of thrombospondin 1 (TSP1 or THBS1), a potent endogenous angiogenesis inhibitor, contributes to angiogenic phenotype in NEPC (66). Several reviews have already described the current knowledge and therapeutic development targeting angiogenesis in prostate cancer (61, 67, 68).

## Clinical Correlation of Neuroendocrine Phenotype, Angiogenesis and Prognosis in Prostate Cancer

Several research groups have shown positive correlations between NE marker expression and angiogenesis in prostate tumors. Higher neovascularization and VEGF staining are observed in prostate tumors with more NE tumor cells (16, 69, 70). Grobholz et al. detected NE marker CHGA and angiogenic marker CD34 in 102 prostatectomy prostate tumor specimens. They found that poorer pathological staging correlates with increased neovascularization and stronger NE marker expression (16). Harper et al. found a positive correlation between VEGF and CHGA levels in 45 prostatic carcinoma specimens (67, 70–72). Borre et al. analyzed VEGF and CHGA expression in 221 prostate tumors (62). They found only tumors with strong expression of both VEGF and NE showed significantly poor clinical characteristics such as higher microvessel density, T stage, dedifferentiation, and shorter disease-specific survival.

## Proteins and Pathways Regulating Both Ne Phenotype and Angiogenesis

**Figure 1:**
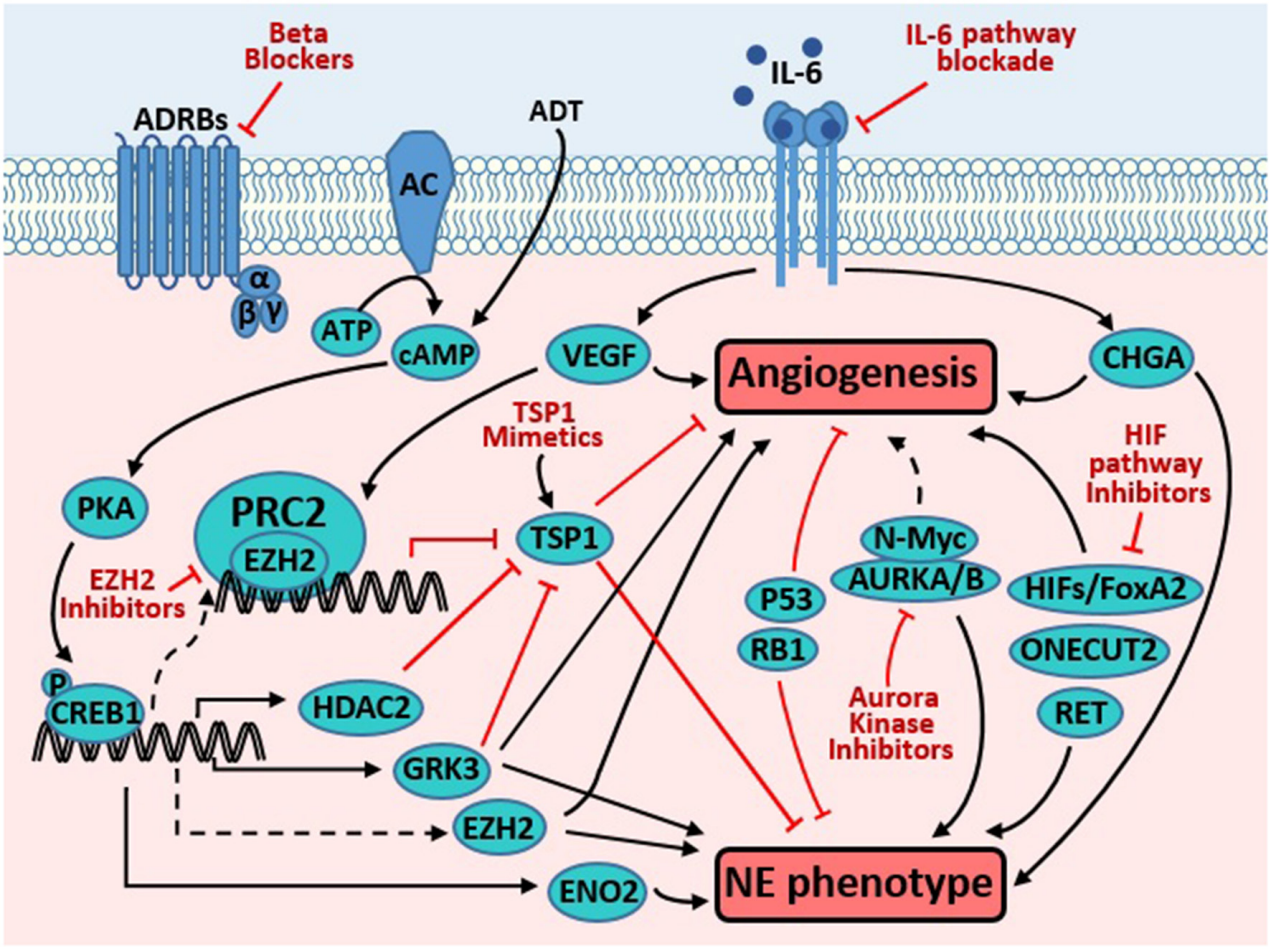
Targeting molecules common to pathways promoting angiogenesis and neuroendocrine phenotype in prostate cancer. Androgen derivation therapy (ADT) elevates cAMP level, which activates PKA, resulting in phosphorylation and activation of CREB1. Activated CREB1 directly induces transcription of several genes involved in neuroendocrine differentiation (NED) and angiogenesis, such as VEGF, ENO2, GRK3, and HDAC2. VEGF is a potent pro-angiogenic factor, while ENO2 is a neuroendocrine marker. GRK3 promotes angiogenesis, NE marker expression, and prostate cancer progression. HDAC2 is critical for prostate cancer progression that is induced by chronical bio-behavioral stress and signals from beta adrenergic receptors (ADRBs). GRK3 and HDAC2 promotes angiogenesis, at least in part through downregulating TSP1. TSP1 is well-established as an anti-angiogenesis factor. Through unclear mechanisms, CREB1 activation enhances the PRC2 function of EZH2, which is critical for NED and angiogenesis induced by ADT. In endothelial cells, VEGF induces EZH2 expression and activity, which contributes to VEGF's action in promoting angiogenesis. Loss of p53 and RB1, alone or in cooperation, promote angiogenesis and NE phenotype through multiple mechanisms (detailed in text). IL-6 pathway activation enhances angiogenesis (through inducing VEGF) and NE phenotype (through inducing CHGA). AURKA interacts with N-Myc and regulates the stability of the latter, which promotes NED. AURKA and AURKB regulate angiogenesis in endothelial and neuroblastoma cells. HIF1A promotes angiogenesis through inducing VEGF. Moreover, it also cooperates with FoxA2 to promote NED and tumorigenesis. ONECUT2 has recently emerged as a master regulator of NED. Recent studies have also implicated receptor tyrosine kinase RET in regulating NED and angiogenesis. Novel strategies targeting the proteins and pathways that regulate both prominent phenotypes may be effective to treat NEPC (detailed in text).

It remains largely unclear whether neuroendocrine differentiation and angiogenesis regulate each other in NEPC. It is also unclear what proteins directly link these two prominent characteristics of NEPC. Our literature search did not yield reports showing direct involvement of pro-angiogenic factors VEGF and neurosecretory peptides (serotonin, bombesin, and gastrin) in promoting NE marker expression. On the other hand, among the NE marker proteins, only CHGA (73, 74) has been shown to directly contribute to angiogenesis. As summarized below and depicted in Figure 1, several signaling proteins have been reported to regulate both angiogenesis and NE marker expression, often in separate diseases or biological contexts. These proteins are potential molecular links between the two important characteristics of NEPC.

### CHGA

CHGA is one of the classic markers for NEPC. It is a secreted glycoprotein that shows paradoxical properties in angiogenesis (71, 73–75). Recent studies showed CHGA can be proteolytically cleaved into active peptides by thrombin. This cleavage shifts its function from anti- to pro-angiogenesis under pathophysiologic conditions, which could be observed in prothrombin activation or multiple myeloma (73, 74). Its function in angiogenesis in NEPC is still unclear.

### p53 and RB1

p53 and RB1, two most prominent tumor suppressors, have been implicated in both angiogenesis and NE marker expression in separate studies. Mutations and loss of p53 or RB1 are common alterations in prostate cancer patients (76). Tumors containing p53 mutations are usually more vascularized than tumors harboring wild type p53. This pattern has been observed in several independent clinical studies on prostate, colon, and breast cancers (77–80). Some basic mechanisms underlying p53's inhibition of angiogenesis have been detailed. Ravi et al. found that, under hypoxic conditions, p53 inhibits the HIF1A activity that is required for VEGF transcription (81). Besides VEGF, p53 also inhibits other pro-angiogenetic factors, such as bFGF-BP (bFGF-binding protein) and COX-2 (cyclooxygenase-2). In addition, p53 also induces anti-angiogenetic factors, such as TSP-1 and EPHA2 (ephrin receptor A2) (82). However, it is not clear whether or how p53 itself plays a role in regulating NE phenotype.

RB1 has also been reported to regulate tumor angiogenesis (83–85). For example, Lasorella et al. reported Id2 (inhibitor of differentiation 2), a target of RB1, mediates angiogenesis of pituitary tumors from *Rb1*^+/−^ mice (86). RB1 loss is one of the most critical events in neuroendocrine carcinoma (12, 87, 88), but the mechanism by which RB1 contributes to NE phenotype is largely unclear. A recent study reported RB1 takes part in regulating both angiogenesis and NE phenotypes. Labrecque et al. found, under hypoxic conditions, RB1-loss deregulates the expression of genes that govern angiogenesis, metastasis and NE differentiation. These effects led to a more invasive phenotype as well as NE protein markers expression in human prostate cancer cells (40).

Growing evidence implies a cooperative function of p53 and RB1 in tumor angiogenesis. Martinez-Cruz et al. found that combinatorial deletion of p53 and RB1 augmented tumor angiogenesis in a spontaneous squamous cell carcinoma mouse model, comparing with loss of p53 alone (89). Similarly, inactivation of p53 and RB1 leads to a pro-angiogenic transcriptional response in keratinocytes (90). In a xenograft model of retinoblastoma, p53 was shown to increase VEGF expression and promote angiogenesis in cells deficient for p21/RB1 pathway (91). All these observations underline the possibility of p53 and RB1 cooperation in promoting prostate cancer angiogenesis.

Interestingly, p53 and RB1 are also both connected to NE marker expression in prostate cancer. In a NEPC xenograft model LTL-331R that relapsed upon castration resistance of prostate adenocarcinoma patient-derived xenograft LTL-331, genomic alterations of both p53 and RB1 were observed (39). Of note, Beltran et al. showed (25) that “concurrent loss of RB1 and p53 was present in 53.3% of NEPC patient tumors vs. 13.7% of CRPC-adenocarcinoma samples (*P* < 0.0004, proportion test).” In a classic NEPC genetically engineered mouse (GEM) model called TRAMP, p53 and RB1 are both inactivated in the prostate by SV40 large T antigen oncoprotein, which induces the development of prostate cancers that subsequently progress to NEPC (92). Using GEM model and human cell models, loss of p53 and RB1 has been shown to promote linear plasticity and a phenotypic shift from AR-dependent luminal epithelial cells to AR-independent NEPC with resistance to enzalutamide (an antiandrogen drug) (26, 36).

### PKA-CREB1 Axis

Both angiogenesis and NE marker expression can be induced by increased cellular cAMP level (93–95). Androgen deprivation therapy (ADT) increases cAMP level in prostate cancer cells, which activates the PKA-CREB1 pathway that in turn regulates both phenotypes. VEGF and ENO2 have been identified as targets of CREB1 and regulate angiogenesis and NE marker expression, respectively (96–98). However, targets of CREB1 that regulate both phenotypes were largely unknown. We recently reported two direct targets of CREB1, GRK3 (G protein coupled receptor kinase 3) and HDAC2 (histone deacetylase 2). GRK3 was shown to promote both angiogenesis and NE marker expression in separate studies (detailed below). Induction of HDAC2 by CREB1 is critical for prostate cancer progression promoted by chronical bio-behavioral stress that activates PKA-CREB1 pathway though beta adrenergic signaling (99). It is still unknown whether HDAC2 is involved in NE phenotype regulation in prostate cancer. In another study, we found that PKA-CREB1 signaling enhances the epigenetic repressive activity of EZH2 (enhanced zeste homolog 2) that in turn induces NE phenotype and angiogenesis (detailed below). In short, the PKA-CREB1 axis seems to be a master upstream regulator for both NE phenotype and angiogenesis in prostate cancer.

### GRK3

We initially uncovered GRK3 as a key regulator of the progression of prostate cancer through unbiased shRNA and focused cDNA screening of human kinases (100). GRK3 is essential for metastatic prostate cancer cells in culture and in mouse xenografts. Further, its overexpression promotes orthotropic prostate tumor growth in mouse xenografts. Mechanistically, GRK3 promotes prostate cancer progression in part through repressing two anti-angiogenic factors TSP1 and PAI2, thus inducing angiogenesis in prostate cancer cells (100). Genomic profiling and immunohistochemical staining of human prostate cancers showed that GRK3 is upregulated in advanced prostate cancers (100, 101). Of note, we found a strong trend between GRK3 protein level and glomeruloid microvascular proliferation, a marker of VEGFA–driven angiogenesis, in prostate cancer patient samples. This result further supports a role of GRK3 in stimulating angiogenesis.

We recently reported that GRK3 promotes ADT resistance and NE marker expression of prostate adenocarcinoma cells (101). The kinase dead form of GRK3 abolished these phenotypes, indicating a requirement of GRK3's kinase activity (100, 101). Moreover, GRK3 is positively associated with NE marker expression in human cancer cell lines and patient tumors. Upon GRK3 silencing, expression of NE markers induced by ADT was reduced. These results suggest that GRK3 is a key regulator of both NE phenotype and angiogenesis in prostate cancer. It is worth further investigating the molecular mechanisms of GRK3 and the potential of inhibiting GRK3 as a novel strategy to block NEPC.

### EZH2

Polycomb repressive complex 2 (PRC2) is another important regulator for both angiogenesis and NEPC. PRC2 usually renders transcriptional repression by tri-methylating lysine 27 of histone H3 (H3K27me3) on target genes (102, 103). As the key catalytic subunit of PRC2, EZH2 is widely overexpressed in many tumors, including prostate cancer (102). Overexpression of EZH2 and elevated PRC2 activity promote prostate cancer cell proliferation and migration (103). Clermont et al. found that EZH2 is one of the most upregulated epigenetic regulators in NEPC across multiple datasets from clinical to xenograft tissues (104). Dardenne et al. reported that high catalytic activity of EZH2 promotes N-Myc-AR-PRC2 complex formation and promotes NE phenotype (37). Ku et al. emphasized that overexpressed EZH2 in prostate-specific *Pten*-*Rb1*-*p53* triple knockout mice plays a pivotal role in promoting prostate cancer lineage plasticity, antiandrogen resistance, and neuroendocrine phenotype (26). We recently demonstrated that EZH2 presents a critical molecular link for NE phenotype and angiogenesis, downstream of ADT-activated PKA-CREB1 signaling (66). EZH2 is activated by ADT and PKA-CREB1 signaling, which in turn induces NE markers and reduces TSP1 in prostate cancer cells. Our study also fills in a gap of knowledge how EZH2 overexpression in cancer cells directly contributes to tumor angiogenesis. Lu et al. have showed that EZH2 is induced by VEGF in endothelial cells, which contributes to angiogenesis (105).

### TSP1

TSP1 is found to have various specific biological activities in different specific tumor environments. The role, regulation and expression patterns of TSP1 in human malignancies are highly context dependent and complicated. On general knowledge of TSP1 in urological cancers, please refer to this outstanding review (106). TSP1 is the first identified endogenous inhibitor of angiogenesis. It suppresses endothelial cell proliferation, migration, and tube formation, as well as induces endothelial apoptosis (107–109). While TSP1's role in angiogenesis is well-known, we recently established its role and regulation in NEPC (66). As expected, TSP1 inhibits angiogenesis induced by NEPC cells. Furthermore, the expression of TSP1 in NEPC is significantly lower than that in CRPC-adenocarcinoma, and NE markers negatively correlate with TSP1 in several prostate cancer datasets (66). Interestingly, TSP1 silence increase NE marker expression in PC3 prostate cancer cells, which suggests that TSP1 may directly regulate NE phenotype. This intriguing observation supports an intimate relation between NE phenotype and angiogenesis in prostate cancer cells (66). The molecular mechanisms underlying TSP1's role of NE phenotype warrants further investigation.

### IL-6

As a pro-inflammatory cytokine, interleukin-6 (IL-6) is expressed in both of prostate tumors and the stromal tumor microenvironment. IL-6 is well-known to participate in cellular angiogenesis. Recently, Culig and Puhr have elegantly reviewed the role and regulation of IL-6 in prostate cancer (110). Several signaling pathways downstream of IL-6 orchestrate angiogenesis and NE phenotype in prostate cancer. For example, Ishii et al. showed that IL-6 promotes angiogenesis by up-regulating VEGF through PI3K/AKT pathway (111). On the other hand, IL-6 boosts NE phenotype by inducing CHGA and ENO2 expression through JAK/STAT3 and MAPK pathways (112, 113), as well as AMPK activation and autophagy induction (114). Detailed molecular mechanisms that connect IL-6 induced angiogenesis and NE phenotype need to be further elucidated.

### MYCN

As a key oncogene driver in neuroblastoma, MYCN (N-Myc) is also a critical regulator of NEPC and SCLC (small cell lung cancer, a poorly differentiated neuroendocrine lung cancer) (21, 37, 71, 115). While convincing evidence supporting a direct role of N-Myc in regulating angiogenesis is scarce, NDRG1 (N-Myc downstream-regulated gene 1) has demonstrated pleiotropic roles in angiogenesis and cancer progression, depending on cancer types (71, 116).

### Aurora Kinases A and B

Aurora kinase A and B (AURKA/B) phosphorylate and stabilize N-Myc protein, which sustains N-Myc function in promoting NE phenotypes in neuroblastoma (117). AURKA and AURKB have been shown to regulate VEGF production and angiogenesis in endothelial cells directly and in neuroblastoma cells (118, 119). It is postulated that AURKA and/or AURKB may regulate angiogenesis of NEPC, although direct evidences are needed.

### HIF1A-FOXA2 Axis

HIF1 and HIF2 are well-known key regulators of angiogenesis (48, 50, 120). Recent studies have also implicated them, especially HIF1A, in regulating neuroendocrine phenotype in prostate cancer. HIF1A cooperates with FOXA2, a transcription factor expressed in NE tissue, to induce several HIF1A target genes that are required for hypoxia-mediated NE phenotype and metastasis in prostate cancer (41, 43).

### ONECUT2 (OC2)

According to recent reports by Rotinen et al. and Guo et al., ONECUT2 plays a critical role in poorly differentiated neuroendocrine prostate tumors as a master transcriptional regulator (41, 121). As a survival factor in mCRPC models, ONECUT2 depresses AR transcription-related program and activates NE differentiation genes and progression to lethal disease (121). Besides, overexpression of ONECUT2 in prostate adenocarcinoma under hypoxia condition is able to inhibit AR signaling and induce NE phenotype (41). Given the crucial role of hypoxia in angiogenesis, we postulate that ONECUT2 may also contribute to the angiogenic phenotype of NEPC, which warrants further study. One study in ovarian cancer demonstrated that silencing ONECUT2 reduces VEGF expression and vascularization in xenograft tumors (122).

### RET

RET mutations are found to enrich in lung adenocarcinoma with NE differentiation (123, 124). Knockdown of RET inhibits prostate tumor growth *in vivo* (125). A recent study from Justin Drake's lab has showed that RET phosphopeptides and mRNA levels are higher in NEPC than in prostate adenocarcinoma, while RET inhibitor AD80 blocks NEPC cell growth in culture and in mouse xenografts (126). Further experiments on gain and loss of function of RET protein will need to be carried out in NEPC cell models. While a role of RET in angiogenesis is well-established in medullary thyroid cancers (127), it is still unclear whether it is critical for the angiogenic phenotype in poorly differentiated neuroendocrine tumors, such as NEPC.

## Targeting the Molecular Links Between Angiogenesis and Ne Phenotype For Developing New Therapies

As summarized above, elevated angiogenesis and NE marker expression are two important interconnected phenotypes. Targeting key molecules linking these two phenotypes may be effective therapeutic strategies for neuroendocrine prostate cancers. Potential therapeutic agents targeting some of these molecules include beta blockers inhibiting PKA-CREB1 signaling, TSP1 mimetic peptides, inhibitors of EZH2 and HIF1 pathway, and IL-6 pathway blockade. It is paramount to evaluate these and other related agents, alone and in combinations, for NEPC, given the reported contributions of their targets in this lethal variant of prostate cancer that has no effective treatment.

### Beta Blockers

Beta blockers which inhibit beta adrenergic signaling and PKA-CREB1 activation, have been used to treat patients with cardiovascular diseases for decades. According to epidemiology studies, cancer patients who have used beta blockers for cardiovascular diseases have better clinical outcomes than the matched patients who do not use, in multiple cancer types, including melanoma, prostate, lung, and breast cancers (128–130). Results from these retrospective investigations are consistent with emerging evidences supporting anti-tumor effects of beta blockers in cancer cells *in vitro* and in mouse xenografts (99, 131–133). Because that beta blockers have been already applied in hypertension and heart diseases for years, they may also become efficient and safe therapies for NEPC. Beta blockers propranolol and carvedilol are tested in several cancer clinical trials (clinicaltrials.gov). However, major obstacles of beta blockers in clinical studies include incomplete understandings of their mechanisms of action in cancers, as well as a shortage of biomarkers for patient selection and efficacy monitoring (129, 134). We recently reported that propranolol downregulates NE marker expression and inhibits angiogenesis and growth of NEPC cell-derived xenografts by blocking a critical pathway CREB1-EZH2-TSP1 (66). This finding suggests that this pathway's activity level may serve as a biomarker for future cancer clinical trials of beta blockers. The therapeutic value of propranolol and other PKA-CREB1 signaling inhibitors in prostate cancer treatment should be further tested.

### EZH2 Inhibitors

Based on the driving role and significant overexpression of EZH2 in many tumors, several inhibitors targeting EZH2 have been developed, such as GSK126, GSK343, GSK503, EPZ6438, CPI-1205, PF-06821497, and DZNeP. Some of these EZH2 inhibitors have demonstrated anti-tumor activity against NEPC *in vitro* and *in vivo*. Beltran et al. found that GSK343 preferentially inhibited cell viability of NEPC cells, while minimally affecting non-NEPC cells (25). Ku et al. reported GSK503 restored enzalutamide sensitivity of prostate tumors from castrated *Pten-Rb1* double knockout mouse (26). DZNeP has also shown some anti-tumor activity in preclinical studies of several cancer types, including prostate cancer (135, 136). We recently demonstrated that conditioned media from prostate cancer cells expressing EZH2 shRNA or treated with GSK126 or EPZ6438 inhibit *in vitro* angiogenesis of endothelial cells (66). In addition, GSK126 and DZNeP were shown to decrease NE marker expression (66). Several EZH2 inhibitors are currently in clinical trials for multiple types of lymphoma, synovial sarcoma, and solid epithelial tumors: NCT03010982 and NCT01897571 (EPZ6438), NCT03480646 (CPI-1205), and NCT03460977 (PF-06821497). It is conceivable that the existing EZH2 inhibitors or other new drugs under development may have positive efficacy targeting NEPC.

### HIF Pathway Inhibitors

Pathways of hypoxia-inducible factors (HIF) play key roles in development of resistance to different treatment modalities. Thereby, HIF pathway inhibitors targeting advanced cancers warrant further clinical studies either as a single agent or in combination with other therapeutic agents (137). Specifically for prostate cancer, two mCRPC clinical trials of HIF pathway inhibitors, including 2ME2 nanocrystal dispersion (panzem) and 17-AAG (tanespimycin), have been reported, which unfortunately showed little efficacy (138, 139). However, given the critical roles of HIF in control both angiogenesis and neuroendocrine phenotypes in NEPC, future testing of other inhibitors of HIF pathway, alone or in combinations, is still justifiable for NEPC. Interestingly, Guo et al. recently showed that TH-302 (evofosfamide), a prodrug activated by hypoxia, significantly inhibits NEPC tumor growth (41). An ongoing immunotherapy study combines ipilimumab (targeting CTLA-4) and evofosfamide for the treatment several solid tumor types, including confirmed metastatic or locally advanced prostate cancers (NCT03098160).

### Aurora Kinase Inhibitor

Phase II trial of Alisertib (MLN8237), an Aurora Kinase A inhibitor, for castration resistant and neuroendocrine prostate cancers was recently completed (140). Although the report did not meet its primary endpoint of significantly extending 6-month radiographic progression-free survival (rPFS), a subset of advanced prostate cancer patients with AURKA and N-Myc activation achieved significant clinical benefits.

### TSP-1 Mimetic Peptides

ABT-510, a TSP-1 mimetic peptide, has been tested in phase I and II clinical trials for many cancer types, including soft tissue sarcoma, metastatic melanoma, renal cell carcinoma, and advanced solid tumors (141–144). ABT-510 failed to show significant clinical benefits as a single agent, suggesting a combinatory strategy is needed. Combination of ABT-510 and cytoxan leads to a delay in progression of PC-3 tumor xenografts (145). Notably, in a phase I study of glioblastoma, combination of ABT-510 with temozolomide and radiotherapy moderately extended overall survival time (146). These findings suggest that combination of ABT-510 with other standard anti-tumor therapies may be an effective strategy to yield better clinical efficacy. Recently, a new TSP-1 mimetic peptide, ABT-898, with greatly increased potency over ABT-510, has been generated. Its efficacy has been tested in rodents and dogs (147–149), and have showed more notable antiangiogenic efficacy than ABT-510 (147). Investigation of the therapeutic potential of ABT-510 and ABT-898 in prostate cancers, especially in NEPC, warrants additional studies.

### IL-6 Pathway Blockade

Given its critical contributions to cancer progression, IL-6 signaling pathway (IL6-/IL6R/JAK/STAT3) is being actively pursued for novel cancer therapies. Recent progress and obstacles in targeting IL-6 to treat cancers have been well-summarized (150–152). Agents blocking IL-6/IL-6R or inhibiting JAK/STAT3 to block tumor progression have been or are being tested in clinical trials, such as siltuximab (an anti-IL-6 mAb), tocilizumab (an anti-IL-6R mAb), Ruxolitinib (a JAK signaling inhibitor). Although many evidences confirmed a key role of IL-6 cascades in regulating the growth of malignant cells in preclinical studies, anti-IL6 or anti-IL6R mAbs have not demonstrated clinical efficacy in several cancer types. The lack of efficacy of IL-6 pathway blockade in cancer is likely due to tumor cells plasticity, displaying different tumor clones in tumor samples *in vivo* (153). Testing IL-6 pathway inhibitors, in combination with standard or other targeted therapies, is still favored for NEPC.

## Future Directions

Besides the knowledge gaps and future directions abovementioned for individual regulators or therapeutic developments, we believe that the following three directions warrant further investigation to fully understand and target the molecules common to pathways promoting angiogenesis and neuroendocrine differentiation of prostate cancer.

### Do Neuroendocrine Differentiation and Angiogenesis Promote Each Other?

We have described several genes reported to regulate both neuroendocrine and angiogenic phenotypes. Much of the knowledge for both phenotypes was in different biological contexts or cancer types. It is largely unclear whether induction of one phenotype leads to increase in another phenotype in the same biological system, such as in NEPC. It is conceivable that induction of neuroendocrine phenotype may promote angiogenesis, in part due to secretion of pro-angiogenic factors by neuroendocrine cells, such as VEGF and neuropeptides bombesin and gastrin, although the roles of these factors in neuroendocrine phenotype are still unclear (15).

### Do Critical Regulators Established in One Phenotype Contribute to the Other Phenotype?

This review mainly focuses on genes that have been implicated in regulating both angiogenesis and neuroendocrine differentiation, although in separate contexts for many genes. To better understand these two phenotypes and to facilitate the development of effective treatments for NEPC, a systematic investigation is necessary to define the roles of these regulators in a shared context. Moreover, studies have characterized the function of several other proteins in regulating either neuroendocrine differentiation (such as BRN2, PEG10, SRRM4, REST, and DEK) or angiogenesis (such as FGF, TGF, EGFL6, PDGF, MMPs, and CCL2). Given the intimate links between the two characteristics as we summarize, it is worthwhile to investigate the roles of critical regulators of neuroendocrine differentiation in regulating angiogenesis, and vice versa.

### Anti-angiogenesis Therapy and Combination Treatments for NEPC?

Positive results in anti-angiogenic therapy were observed in pancreatic neuroendocrine tumors (PNET), another type of neuroendocrine tumors that are well-differentiated with better prognosis than SCLC and NEPC. Sunitinib is a multi-targeted tyrosine kinase receptor inhibitor of VEGFR1-3, PDGFR, c-kit, RET, CSF-1R, and FLT3. It has demonstrated direct antitumor and antiangiogenic effects, and has received FDA approval for the treatment of locally advanced or metastatic PNETs (154, 155).

In SCLC, it was demonstrated that higher VEGF is associated with poor prognosis, which makes it a reasonable strategy to block VEGF pathway for inhibiting angiogenesis and tumor progression. However, only limited clinical benefits in this attempt was observed (156). As far as we know, no result has been reported on clinical trials of anti-angiogenic therapy for NEPC. Due to the striking pathological similarity between SCLC and NEPC, it is likely that, for NEPC, finding the right combinations of anti-angiogenesis and other therapies will be key to achieve significant efficacy for NEPC. Several strategies of combining anti-angiogenic regimens with targeted/chemo/immune therapies have been or are being tested clinically in several cancer types (59). These strategies include combining different anti-angiogenic regimens, simultaneously inhibiting angiogenesis and driving oncogenes, or combining anti-angiogenic regimens with immunotherapy. It is conceivable that similar combinatorial strategies are applicable to NEPC.

Another strategy for NEPC is to target key regulators for both NEPC phenotypes that we have discussed, i.e., neuroendocrine differentiation and angiogenesis, hitting two birds with one stone. In section Targeting the Molecular Links Between Angiogenesis and NE Phenotype for Developing New Therapies, we have summarized some opportunities for developing therapeutics to target pathways involved in both angiogenesis and neuroendocrine phenotypes. It may be necessary to co-target multiple key regulators of both phenotypes to simultaneously block alternative pathways that NEPC cells may use to escape.

## Conclusion

NEPC is lethal without effective treatment. It is still poorly understood. They often have both elevated neuroendocrine marker expression and increased angiogenesis, the mechanisms of which remain largely elusive. Here, we summarize the literature on several proteins and pathways that regulate both angiogenesis and neuroendocrine phenotype in prostate cancer and other contexts. Bridging the mechanistic gaps between regulation of angiogenesis and neuroendocrine phenotype will facilitate better understanding of NEPC progression. We also discuss the opportunities of targeting some of these key regulators to inhibit both angiogenesis and neuroendocrine phenotype for treatments of patients with NEPC. Furthermore, many of the molecular mechanisms that we discuss here for NEPC are also dysregulated in small cell lung cancer (SCLC), a poorly differentiated aggressive neuroendocrine lung carcinoma. Therefore, we expect that much of the current knowledge and new therapeutic potentials summarized here for NEPC are relevant to SCLC.

## Author Contributions

ZW, YZ, ZA, and WL wrote the paper.

### Conflict of Interest

The authors declare that the research was conducted in the absence of any commercial or financial relationships that could be construed as a potential conflict of interest.

## References

[1] Agoulnik IU Vaid A Nakka M Alvarado M Bingman WE III Erdem H. Androgens modulate expression of transcription intermediary factor 2, an androgen receptor coactivator whose expression level correlates with early biochemical recurrence in prostate cancer. Cancer Res. (2006) 66:10594–602. 10.1158/0008-5472.CAN-06-1023 17079484

[2] Komiya A Yasuda K Watanabe A Fujiuchi Y Tsuzuki T Fuse H. The prognostic significance of loss of the androgen receptor and neuroendocrine differentiation in prostate biopsy specimens among castration-resistant prostate cancer patients. Mol Clin Oncol. (2013) 1:257–62. 10.3892/mco.2013.69 24649157 PMC3915703

[3] Wang Q Li W Zhang Y Yuan X Xu K Yu J. Androgen receptor regulates a distinct transcription program in androgen-independent prostate cancer. Cell. (2009) 138:245–56. 10.1016/j.cell.2009.04.056 19632176 PMC2726827

[4] Zhu ML Kyprianou N. Role of androgens and the androgen receptor in epithelial-mesenchymal transition and invasion of prostate cancer cells. Faseb J. (2010) 24:769–77. 10.1096/fj.09-136994 19901020 PMC2830130

[5] Aparicio A Logothetis CJ Maity SN. Understanding the lethal variant of prostate cancer: power of examining extremes. Cancer Disc. (2011) 1:466–8. 10.1158/2159-8290.CD-11-0259 22586648 PMC4133693

[6] Beltran H Tagawa ST Park K MacDonald T Milowsky MI Mosquera JM. Challenges in recognizing treatment-related neuroendocrine prostate cancer. J Clin Oncol. (2012) 30:e386–9. 10.1200/JCO.2011.41.5166 23169519

[7] Hirano D Okada Y Minei S Takimoto Y Nemoto N. Neuroendocrine differentiation in hormone refractory prostate cancer following androgen deprivation therapy. Eur Urol. (2004) 45:586–92. 10.1016/j.eururo.2003.11.032 15082200

[8] Papandreou CN Daliani DD Thall PF Tu SM Wang X Reyes A. Results of a phase II study with doxorubicin, etoposide, and cisplatin in patients with fully characterized small-cell carcinoma of the prostate. J Clin Oncol. (2002) 20:3072–80. 10.1200/JCO.2002.12.065 12118020

[9] Wang HT Yao YH Li BG Tang Y Chang JW Zhang J. Neuroendocrine prostate cancer (NEPC) progressing from conventional prostatic adenocarcinoma: factors associated with time to development of NEPC and survival from NEPC diagnosis-a systematic review and pooled analysis. J Clin Oncol. (2014) 32:3383–90. 10.1200/JCO.2013.54.3553 25225419

[10] Goodman OB Jr Flaig TW Molina A Mulders PF Fizazi K Suttmann H. Exploratory analysis of the visceral disease subgroup in a phase III study of abiraterone acetate in metastatic castration-resistant prostate cancer. Prostate Cancer Prostat Dis. (2014) 17:34–9. 10.1038/pcan.2013.41 24080993 PMC3921671

[11] Scher HI Fizazi K Saad F Taplin ME Sternberg CN Miller K. Increased survival with enzalutamide in prostate cancer after chemotherapy. N Engl J Med. (2012) 367:1187–97. 10.1056/NEJMoa1207506 22894553

[12] Tan HL Sood A Rahimi HA Wang W Gupta N Hicks J. Rb loss is characteristic of prostatic small cell neuroendocrine carcinoma. Clin Cancer Res. (2014) 20:890–903. 10.1158/1078-0432.CCR-13-1982 24323898 PMC3931005

[13] Villaume K Blanc M Gouysse G Walter T Couderc C Nejjari M. VEGF secretion by neuroendocrine tumor cells is inhibited by octreotide and by inhibitors of the PI3K/AKT/mTOR pathway. Neuroendocrinology. (2010) 91:268–78. 10.1159/000289569 20389030

[14] Yazdani S Kasajima A Tamaki K Nakamura Y Fujishima F Ohtsuka H. Angiogenesis and vascular maturation in neuroendocrine tumors. Hum Pathol. (2014) 45:866–74. 10.1016/j.humpath.2013.09.024 24656098

[15] Heinrich E Trojan L Friedrich D Voss M Weiss C Michel MS. Neuroendocrine tumor cells in prostate cancer: evaluation of the neurosecretory products serotonin, bombesin, and gastrin - impact on angiogenesis and clinical follow-up. Prostate. (2011) 71:1752–8. 10.1002/pros.21392 21480309

[16] Grobholz R Bohrer MH Siegsmund M Junemann KP Bleyl U Woenckhaus M. Correlation between neovascularisation and neuroendocrine differentiation in prostatic carcinoma. Pathol Res Pract. (2000) 196:277–84. 10.1016/S0344-0338(00)80056-4 10834383

[17] Beltran H Tomlins S Aparicio A Arora V Rickman D Ayala G. Aggressive variants of castration-resistant prostate cancer. Clin Cancer Res. (2014) 20:2846–50. 10.1158/1078-0432.CCR-13-3309 24727321 PMC4040316

[18] Aggarwal R Huang J Alumkal JJ Zhang L Feng FY Thomas GV. Clinical and genomic characterization of treatment-emergent small-cell neuroendocrine prostate cancer: a multi-institutional prospective study. J Clin Oncol. (2018) 36:2492–503. 10.1200/JCO.2017.77.6880 29985747 PMC6366813

[19] Puca L Vlachostergios PJ Beltran H. Neuroendocrine differentiation in prostate cancer: emerging biology, models, and therapies. Cold Spring Harbor Persp Med. (2019) 9:a030593. 10.1101/cshperspect.a030593 29844220 PMC6360865

[20] Conteduca V Aieta M Amadori D De Giorgi U. Neuroendocrine differentiation in prostate cancer: current and emerging therapy strategies. Crit Rev Oncol Hematol. (2014) 92:11–24. 10.1016/j.critrevonc.2014.05.008 24952997

[21] Beltran H Rickman DS Park K Chae SS Sboner A MacDonald TY. Molecular characterization of neuroendocrine prostate cancer and identification of new drug targets. Cancer Disc. (2011) 1:487–95. 10.1158/2159-8290.CD-11-0130 22389870 PMC3290518

[22] Jongsma J Oomen MH Noordzij MA Van Weerden WM Martens GJ van der Kwast TH. Kinetics of neuroendocrine differentiation in an androgen-dependent human prostate xenograft model. Am J Pathol. (1999) 154:543–51. 10.1016/S0002-9440(10)65300-X 10027412 PMC1850014

[23] Terry S Beltran H. The many faces of neuroendocrine differentiation in prostate cancer progression. Front Oncol. (2014) 4:60. 10.3389/fonc.2014.00060 24724054 PMC3971158

[24] Vlachostergios PJ Papandreou CN. Targeting neuroendocrine prostate cancer: molecular and clinical perspectives. Front Oncol. (2015) 5:6. 10.3389/fonc.2015.00006 25699233 PMC4313607

[25] Beltran H Prandi D Mosquera JM Benelli M Puca L Cyrta J. Divergent clonal evolution of castration-resistant neuroendocrine prostate cancer. Nat Med. (2016) 22:298–305. 10.1038/nm.4045 26855148 PMC4777652

[26] Ku SY Rosario S Wang Y Mu P Seshadri M Goodrich ZW. Rb1 and Trp53 cooperate to suppress prostate cancer lineage plasticity, metastasis, and antiandrogen resistance. Science. (2017) 355:78–83. 10.1126/science.aah4199 28059767 PMC5367887

[27] Zou M Toivanen R Mitrofanova A Floch N Hayati S Sun Y. Transdifferentiation as a mechanism of treatment resistance in a mouse model of castration-resistant prostate cancer. Cancer Dis. (2017) 7:736–49. 10.1158/2159-8290.CD-16-1174 28411207 PMC5501744

[28] Bishop JL Davies A Ketola K Zoubeidi A. Regulation of tumor cell plasticity by the androgen receptor in prostate cancer. Endocr Relat Cancer. (2015) 22:R165–82. 10.1530/ERC-15-0137 25934687

[29] Chen R Dong X Gleave M. Molecular model for neuroendocrine prostate cancer progression. BJU Int. (2018) 122:560–70. 10.1111/bju.14207 29569310

[30] Davies AH Beltran H Zoubeidi A. Cellular plasticity and the neuroendocrine phenotype in prostate cancer. Nat Rev Urol. (2018) 15:271–86. 10.1038/nrurol.2018.22 29460922

[31] Soundararajan R Paranjape AN Maity S Aparicio A Mani SA. EMT, stemness and tumor plasticity in aggressive variant neuroendocrine prostate cancers. Biochim Biophys Acta Rev Cancer. (2018) 1870:229–38. 10.1016/j.bbcan.2018.06.006 29981816 PMC6496942

[32] Hu CD Choo R Huang J. Neuroendocrine differentiation in prostate cancer: a mechanism of radioresistance and treatment failure. Front Oncol. (2015) 5:90. 10.3389/fonc.2015.00090 25927031 PMC4396194

[33] Li Y Donmez N Sahinalp C Xie N Wang Y Xue H. SRRM4 drives neuroendocrine transdifferentiation of prostate adenocarcinoma under androgen receptor pathway inhibition. Eur Urol. (2016) 71:68–78. 10.1016/j.eururo.2016.04.028 27180064

[34] Zhang X Coleman IM Brown LG True LD Kollath L Lucas JM. SRRM4 expression and the loss of REST activity may promote the emergence of the neuroendocrine phenotype in castration-resistant prostate cancer. Clin Cancer Res. (2015) 21:4698–708. 10.1158/1078-0432.CCR-15-0157 26071481 PMC4609255

[35] Lin D Dong X Wang K Wyatt AW Crea F Xue H. Identification of DEK as a potential therapeutic target for neuroendocrine prostate cancer. Oncotarget. (2015) 6:1806–20. 10.18632/oncotarget.2809 25544761 PMC4359333

[36] Mu P Zhang Z Benelli M Karthaus WR Hoover E Chen CC. SOX2 promotes lineage plasticity and antiandrogen resistance in TP53- and RB1-deficient prostate cancer. Science. (2017) 355:84–8. 10.1126/science.aah4307 28059768 PMC5247742

[37] Dardenne E Beltran H Benelli M Gayvert K Berger A Puca L. N-Myc induces an EZH2-mediated transcriptional program driving neuroendocrine prostate cancer. Cancer Cell. (2016) 30:563–77. 10.1016/j.ccell.2016.09.005 27728805 PMC5540451

[38] Bishop JL Thaper D Vahid S Davies A Ketola K Kuruma H. The master neural transcription factor BRN2 is an androgen receptor-suppressed driver of neuroendocrine differentiation in prostate cancer. Cancer Disc. (2017) 7:54–71. 10.1158/2159-8290.CD-15-1263 27784708

[39] Akamatsu S Wyatt AW Lin D Lysakowski S Zhang F Kim S. The placental gene PEG10 promotes progression of neuroendocrine prostate cancer. Cell Rep. (2015) 12:922–36. 10.1016/j.celrep.2015.07.012 26235627

[40] Labrecque MP Takhar MK Nason R Santacruz S Tam KJ Massah S. The retinoblastoma protein regulates hypoxia-inducible genetic programs, tumor cell invasiveness and neuroendocrine differentiation in prostate cancer cells. Oncotarget. (2016) 7:24284–302. 10.18632/oncotarget.8301 27015368 PMC5029701

[41] Guo H Ci X Ahmed M Hua JT Soares F Lin D. ONECUT2 is a driver of neuroendocrine prostate cancer. Nat Commun. (2019) 10:278. 10.1038/s41467-018-08133-6 30655535 PMC6336817

[42] Park JW Lee JK Witte ON Huang J. FOXA2 is a sensitive and specific marker for small cell neuroendocrine carcinoma of the prostate. Modern Pathol. (2017) 30:1262–72. 10.1038/modpathol.2017.44 28621319 PMC6330177

[43] Qi J Nakayama K Cardiff RD Borowsky AD Kaul K Williams R. Siah2-dependent concerted activity of HIF and FoxA2 regulates formation of neuroendocrine phenotype and neuroendocrine prostate tumors. Cancer Cell. (2010) 18:23–38. 10.1016/j.ccr.2010.05.024 20609350 PMC2919332

[44] Mirosevich J Gao N Gupta A Shappell SB Jove R Matusik RJ. Expression and role of Foxa proteins in prostate cancer. Prostate. (2006) 66:1013–28. 10.1002/pros.20299 16001449

[45] Risau W. Mechanisms of angiogenesis. Nature. (1997) 386:671–4. 10.1038/386671a0 9109485

[46] Burri PH Djonov V. Intussusceptive angiogenesis–the alternative to capillary sprouting. Mol Aspects Med. (2002) 23:S1–27. 10.1016/S0098-2997(02)00096-1 12537983

[47] Carmeliet P. Angiogenesis in life, disease and medicine. Nature. (2005) 438:932–6. 10.1038/nature04478 16355210

[48] Carmeliet P Jain RK. Angiogenesis in cancer and other diseases. Nature. (2000) 407:249–57. 10.1038/35025220 11001068

[49] Marech I Leporini C Ammendola M Porcelli M Gadaleta CD Russo E. Classical and non-classical proangiogenic factors as a target of antiangiogenic therapy in tumor microenvironment. Cancer Lett. (2016) 380:216–26. 10.1016/j.canlet.2015.07.028 26238184

[50] Krock BL Skuli N Simon MC. Hypoxia-induced angiogenesis: good and evil. Genes Cancer. (2011) 2:1117–33. 10.1177/1947601911423654 22866203 PMC3411127

[51] Rundhaug JE Matrix metalloproteinases and angiogenesis. J Cell Mol Med. (2005) 9:267–85. 10.1111/j.1582-4934.2005.tb00355.x 15963249 PMC6740080

[52] An J Du Y Fan X Wang Y Ivan C Zhang XG. EGFL6 promotes breast cancer by simultaneously enhancing cancer cell metastasis and stimulating tumor angiogenesis. Oncogene. (2019) 38:2123–34. 10.1038/s41388-018-0565-9 30455428 PMC6579606

[53] Ridiandries A Tan JT Bursill CA. The role of CC-chemokines in the regulation of angiogenesis. Int J Mol Sci. (2016) 17:1856. 10.3390/ijms17111856 27834814 PMC5133856

[54] Fagiani E Christofori G. Angiopoietins in angiogenesis. Cancer Lett. (2013) 328:18–26. 10.1016/j.canlet.2012.08.018 22922303

[55] Bagnato A Spinella F. Emerging role of endothelin-1 in tumor angiogenesis. Trends Endocrinol Metab. (2003) 14:44–50. 10.1016/S1043-2760(02)00010-3 12475611

[56] Guerrero PA McCarty JH TGF-β Activation and Signaling in Angiogenesis. London, UK: IntechOpen (2017). 10.5772/66405

[57] Al-Abd AM Alamoudi AJ Abdel-Naim AB Neamatallah TA Ashour OM. Anti-angiogenic agents for the treatment of solid tumors: potential pathways, therapy and current strategies - a review. J Adv Res. (2017) 8:591–605. 10.1016/j.jare.2017.06.006 28808589 PMC5544473

[58] Yadav L Puri N Rastogi V Satpute P Sharma V. Tumour angiogenesis and angiogenic inhibitors: a review. J Clin Diagn Res. (2015) 9:XE01–5. 10.7860/JCDR/2015/12016.6135 26266204 PMC4525594

[59] Comunanza V Bussolino F. Therapy for cancer: strategy of combining anti-angiogenic and target therapies. Front Cell Dev Biol. (2017) 5:101. 10.3389/fcell.2017.00101 29270405 PMC5725406

[60] Noh K Mangala LS Han HD Zhang N Pradeep S Wu SY. Differential effects of EGFL6 on tumor versus wound angiogenesis. Cell Rep. (2017) 21:2785–95. 10.1016/j.celrep.2017.11.020 29212026 PMC5749980

[61] Li Y Cozzi PJ. Angiogenesis as a strategic target for prostate cancer therapy. Med Res Rev. (2010) 30:23–66. 10.1002/med.20161 19536866

[62] Borre M Offersen BV Nerstrom B Overgaard J. Microvessel density predicts survival in prostate cancer patients subjected to watchful waiting. Br J Cancer. (1998) 78:940–4. 10.1038/bjc.1998.605 9764587 PMC2063118

[63] Bono AV Celato N Cova V Salvadore M Chinetti S Novario R. Microvessel density in prostate carcinoma. Prostate Cancer Prostat Dis. (2002) 5:123–7. 10.1038/sj.pcan.4500572 12497001

[64] Strohmeyer D Rossing C Strauss F Bauerfeind A Kaufmann O Loening S. Tumor angiogenesis is associated with progression after radical prostatectomy in pT2/pT3 prostate cancer. Prostate. (2000) 42:26–33. 10.1002/(SICI)1097-0045(20000101)42:1<26::AID-PROS4>3.0.CO;2-6 10579796

[65] Vartanian RK Weidner N. Endothelial cell proliferation in prostatic carcinoma and prostatic hyperplasia: correlation with Gleason's score, microvessel density, and epithelial cell proliferation. Lab Invest. (1995) 73:844–50. 8558846

[66] Zhang Y Zheng D Zhou T Song H Hulsurkar M Su N. Androgen deprivation promotes neuroendocrine differentiation and angiogenesis through CREB-EZH2-TSP1 pathway in prostate cancers. Nat Commun. (2018) 9:4080. 10.1038/s41467-018-06177-2 30287808 PMC6172226

[67] van Moorselaar RJ Voest EE. Angiogenesis in prostate cancer: its role in disease progression and possible therapeutic approaches. Mol Cell Endocrinol. (2002) 197:239–50. 10.1016/S0303-7207(02)00262-9 12431818

[68] Russo G Mischi M Scheepens W De la Rosette JJ Wijkstra H. Angiogenesis in prostate cancer: onset, progression and imaging. BJU Int. (2012) 110:E794–808. 10.1111/j.1464-410X.2012.11444.x 22958524

[69] Revelos K Petraki C Scorilas A Stefanakis S Malovrouvas D Alevizopoulos N. Correlation of androgen receptor status, neuroendocrine differentiation and angiogenesis with time-to-biochemical failure after radical prostatectomy in clinically localized prostate cancer. Anticancer Res. (2007) 27:3651–60. 17972531

[70] Harper ME Glynne-Jones E Goddard L Thurston VJ Griffiths K. Vascular endothelial growth factor (VEGF) expression in prostatic tumours and its relationship to neuroendocrine cells. Br J Cancer. (1996) 74:910–6. 10.1038/bjc.1996.456 8826857 PMC2074752

[71] Bae DH Jansson PJ Huang ML Kovacevic Z Kalinowski D Lee CS. The role of NDRG1 in the pathology and potential treatment of human cancers. J Clin Pathol. (2013) 66:911–7. 10.1136/jclinpath-2013-201692 23750037

[72] Roberts E Cossigny DA Quan GM. The role of vascular endothelial growth factor in metastatic prostate cancer to the skeleton. Prostate Cancer. (2013) 2013:418340. 10.1155/2013/418340 24396604 PMC3874956

[73] Crippa L Bianco M Colombo B Gasparri AM Ferrero E Loh YP. A new chromogranin A-dependent angiogenic switch activated by thrombin. Blood. (2013) 121:392–402. 10.1182/blood-2012-05-430314 23190532 PMC3544118

[74] Bianco M Gasparri AM Colombo B Curnis F Girlanda S Ponzoni M. Chromogranin A is preferentially cleaved into proangiogenic peptides in the bone marrow of multiple myeloma patients. Cancer Res. (2016) 76:1781–91. 10.1158/0008-5472.CAN-15-1637 26869462

[75] Helle KB Corti A. Chromogranin A: a paradoxical player in angiogenesis and vascular biology. Cell Mol Life Sci. (2015) 72:339–48. 10.1007/s00018-014-1750-9 25297920 PMC11113878

[76] Khemlina G Ikeda S Kurzrock R. Molecular landscape of prostate cancer: implications for current clinical trials. Cancer Treat Rev. (2015) 41:761–6. 10.1016/j.ctrv.2015.07.001 26210103

[77] Yu EY Yu E Meyer GE Brawer MK. The relation of p53 protein nuclear accumulation and angiogenesis in human prostatic carcinoma. Prostate Cancer Prostat Dis. (1997) 1:39–44. 10.1038/sj.pcan.4500205 12496932

[78] Takahashi Y Bucana CD Cleary KR Ellis LM. p53, vessel count, and vascular endothelial growth factor expression in human colon cancer. Int J Cancer. (1998) 79:34–38. 10.1002/(SICI)1097-0215(19980220)79:1<34::AID-IJC7>3.0.CO;2-X 9495355

[79] Gasparini G Weidner N Bevilacqua P Maluta S Dalla Palma P Caffo O. Tumor microvessel density, p53 expression, tumor size, and peritumoral lymphatic vessel invasion are relevant prognostic markers in node-negative breast carcinoma. J Clin Oncol. (1994) 12:454–66. 10.1200/JCO.1994.12.3.454 7509851

[80] Faviana P Boldrini L Spisni R Berti P Galleri D Biondi R. Neoangiogenesis in colon cancer: correlation between vascular density, vascular endothelial growth factor (VEGF) and p53 protein expression. Oncol Rep. (2002) 9:617–20. 10.3892/or.9.3.617 11956638

[81] Ravi R Mookerjee B Bhujwalla ZM Sutter CH Artemov D Zeng Q. Regulation of tumor angiogenesis by p53-induced degradation of hypoxia-inducible factor 1alpha. Genes Dev. (2000) 14:34–44. 10.1101/gad.14.1.34 10640274 PMC316350

[82] Teodoro JG Evans SK Green MR. Inhibition of tumor angiogenesis by p53: a new role for the guardian of the genome. J Mol Med. (2007) 85:1175–86. 10.1007/s00109-007-0221-2 17589818

[83] Gabellini C Del Bufalo D Zupi G. Involvement of RB gene family in tumor angiogenesis. Oncogene. (2006) 25:5326–32. 10.1038/sj.onc.1209631 16936754

[84] Schaal C Pillai S Chellappan SP. The Rb-E2F transcriptional regulatory pathway in tumor angiogenesis and metastasis. Adv Cancer Res. (2014) 121:147–82. 10.1016/B978-0-12-800249-0.00004-4 24889531

[85] Bakker WJ Weijts BG Westendorp B de Bruin A. HIF proteins connect the RB-E2F factors to angiogenesis. Transcription. (2013) 4:62–6. 10.4161/trns.23680 23412359 PMC3646055

[86] Lasorella A Rothschild G Yokota Y Russell RG Iavarone A. Id2 mediates tumor initiation, proliferation, and angiogenesis in Rb mutant mice. Mol Cell Biol. (2005) 25:3563–74. 10.1128/MCB.25.9.3563-3574.2005 15831462 PMC1084294

[87] Yachida S Vakiani E White CM Zhong Y Saunders T Morgan R. Small cell and large cell neuroendocrine carcinomas of the pancreas are genetically similar and distinct from well-differentiated pancreatic neuroendocrine tumors. Am J Surg Pathol. (2012) 36:173–84. 10.1097/PAS.0b013e3182417d36 22251937 PMC3261427

[88] Rickman DS Beltran H Demichelis F Rubin MA. Biology and evolution of poorly differentiated neuroendocrine tumors. Nat Med. (2017) 23:1–10. 10.1038/nm.4341 28586335

[89] Martinez-Cruz AB Santos M Lara MF Segrelles C Ruiz S Moral M. Spontaneous squamous cell carcinoma induced by the somatic inactivation of retinoblastoma and Trp53 tumor suppressors. Cancer Res. (2008) 68:683–92. 10.1158/0008-5472.CAN-07-3049 18245467

[90] Toussaint-Smith E Donner DB Roman A. Expression of human papillomavirus type 16 E6 and E7 oncoproteins in primary foreskin keratinocytes is sufficient to alter the expression of angiogenic factors. Oncogene. (2004) 23:2988–95. 10.1038/sj.onc.1207442 14968115

[91] Farhang Ghahremani M Goossens S Nittner D Bisteau X Bartunkova S Zwolinska A. p53 promotes VEGF expression and angiogenesis in the absence of an intact p21-Rb pathway. Cell Death Differ. (2013) 20:888–97. 10.1038/cdd.2013.12 23449391 PMC3679451

[92] Gingrich JR Barrios RJ Kattan MW Nahm HS Finegold MJ Greenberg NM. Androgen-independent prostate cancer progression in the TRAMP model. Cancer Res. (1997) 57:4687–91. 9354422

[93] Zhang Y Daaka Y. PGE2 promotes angiogenesis through EP4 and PKA Cgamma pathway. Blood. (2011) 118:5355–64. 10.1182/blood-2011-04-350587 21926356 PMC3217416

[94] Garg J Feng YX Jansen SR Friedrich J Lezoualc'h F Schmidt M. Catecholamines facilitate VEGF-dependent angiogenesis via beta2-adrenoceptor-induced Epac1 and PKA activation. Oncotarget. (2017) 8:44732–48. 10.18632/oncotarget.17267 28512254 PMC5546514

[95] Bang YJ Pirnia F Fang WG Kang WK Sartor O Whitesell L. Terminal neuroendocrine differentiation of human prostate carcinoma cells in response to increased intracellular cyclic AMP. Proc Natl Acad Sci USA. (1994) 91:5330–4. 10.1073/pnas.91.12.5330 8202489 PMC43988

[96] Jeon SH Chae BC Kim HA Seo GY Seo DW Chun GT. The PKA/CREB pathway is closely involved in VEGF expression in mouse macrophages. Mol Cells. (2007) 23:23–9. 17464208

[97] Rhee SH Ma EL Lee Y Tache Y Pothoulakis C Im E. Corticotropin releasing hormone and urocortin 3 stimulate vascular endothelial growth factor expression through the cAMP/CREB pathway. J Biol Chem. (2015) 290:26194–203. 10.1074/jbc.M115.678979 26350463 PMC4646269

[98] Park MH Lee HS Lee CS You ST Kim DJ Park BH. p21-Activated kinase 4 promotes prostate cancer progression through CREB. Oncogene. (2013) 32:2475–82. 10.1038/onc.2012.255 22710715

[99] Hulsurkar M Li Z Zhang Y Li X Zheng D Li W. Beta-adrenergic signaling promotes tumor angiogenesis and prostate cancer progression through HDAC2-mediated suppression of thrombospondin-1. Oncogene. (2017) 36:1525–36. 10.1038/onc.2016.319 27641328

[100] Li W Ai N Wang S Bhattacharya N Vrbanac V Collins M. GRK3 is essential for metastatic cells and promotes prostate tumor progression. Proc Natl Acad Sci USA. (2014) 111:1521–6. 10.1073/pnas.1320638111 24434559 PMC3910602

[101] Sang M Hulsurkar M Zhang X Song H Zheng D Zhang Y. GRK3 is a direct target of CREB activation and regulates neuroendocrine differentiation of prostate cancer cells. Oncotarget. (2016) 7:45171–85. 10.18632/oncotarget.9359. 27191986 PMC5216714

[102] Varambally S Dhanasekaran SM Zhou M Barrette TR Kumar-Sinha C Sanda MG. The polycomb group protein EZH2 is involved in progression of prostate cancer. Nature. (2002) 419:624–9. 10.1038/nature01075 12374981

[103] Yang YA Yu J. EZH2, an epigenetic driver of prostate cancer. Protein Cell. (2013) 4:331–41. 10.1007/s13238-013-2093-2 23636686 PMC4131440

[104] Clermont PL Lin D Crea F Wu R Xue H Wang Y. Polycomb-mediated silencing in neuroendocrine prostate cancer. Clin Epigenetics. (2015) 7:40. 10.1186/s13148-015-0074-4 25859291 PMC4391120

[105] Lu C Han HD Mangala LS Ali-Fehmi R Newton CS Ozbun L. Regulation of tumor angiogenesis by EZH2. Cancer Cell. (2010) 18:185–97. 10.1016/j.ccr.2010.06.016 20708159 PMC2923653

[106] Miyata Y Sakai H. Thrombospondin-1 in urological cancer: pathological role, clinical significance, and therapeutic prospects. Int J Mol Sci. (2013) 14:12249–72. 10.3390/ijms140612249 23749112 PMC3709784

[107] Taraboletti G Roberts D Liotta LA Giavazzi R. Platelet thrombospondin modulates endothelial cell adhesion, motility, and growth: a potential angiogenesis regulatory factor. J Cell Biol. (1990) 111:765–72. 10.1083/jcb.111.2.765 1696271 PMC2116188

[108] Tolsma SS Stack MS Bouck N. Lumen formation and other angiogenic activities of cultured capillary endothelial cells are inhibited by thrombospondin-1. Microvasc Res. (1997) 54:13–26. 10.1006/mvre.1997.2015 9245640

[109] Jimenez B Volpert OV Crawford SE Febbraio M Silverstein RL Bouck N. Signals leading to apoptosis-dependent inhibition of neovascularization by thrombospondin-1. Nat Med. (2000) 6:41–8. 10.1038/71517 10613822

[110] Culig Z Puhr M. Interleukin-6 and prostate cancer: current developments and unsolved questions. Mol Cell Endocrinol. (2018) 462:25–30. 10.1007/978-1-4939-7845-8 28315704

[111] Ishii K Sasaki T Iguchi K Kajiwara S Kato M Kanda H. Interleukin-6 induces VEGF secretion from prostate cancer cells in a manner independent of androgen receptor activation. Prostate. (2018) 78:849–56. 10.1002/pros.23643 29707793

[112] Spiotto MT Chung TD. STAT3 mediates IL-6-induced neuroendocrine differentiation in prostate cancer cells. Prostate. (2000) 42:186–95. 10.1002/(SICI)1097-0045(20000215)42:3<186::AID-PROS4>3.0.CO;2-E 10639189

[113] Deeble PD Murphy DJ Parsons SJ Cox ME. Interleukin-6- and cyclic AMP-mediated signaling potentiates neuroendocrine differentiation of LNCaP prostate tumor cells. Mol Cell Biol. (2001) 21:8471–82. 10.1128/MCB.21.24.8471-8482.2001 11713282 PMC100010

[114] Chang PC Wang TY Chang YT Chu CY Lee CL Hsu HW. Autophagy pathway is required for IL-6 induced neuroendocrine differentiation and chemoresistance of prostate cancer LNCaP cells. PLoS ONE. (2014) 9:e88556. 10.1371/journal.pone.0088556 24551118 PMC3925144

[115] Nau MM Brooks BJ Jr Carney DN Gazdar AF Battey JF Sausville EA. Human small-cell lung cancers show amplification and expression of the N-myc gene. Proc Natl Acad Sci USA. (1986) 83:1092–6. 10.1073/pnas.83.4.1092 2869482 PMC323017

[116] Park KC Paluncic J Kovacevic Z Richardson DR. Pharmacological targeting and the diverse functions of the metastasis suppressor, NDRG1, in cancer. Free Rad Biol Med. (2019). 10.1016/j.freeradbiomed.2019.05.020 31132412

[117] Otto T Horn S Brockmann M Eilers U Schuttrumpf L Popov N. Stabilization of N-Myc is a critical function of Aurora A in human neuroblastoma. Cancer Cell. (2009) 15:67–78. 10.1016/j.ccr.2008.12.005 19111882

[118] Sun X Niu S Zhang Z Wang A Yang C Guo Z. Aurora kinase inhibitor VX680 suppresses the proliferation and migration of HUVECs and angiogenesis. Mol Med Rep. (2019) 19:3841–7. 10.3892/mmr.2019.9996 30816538

[119] Romain C Paul P Kim KW Lee S Qiao J Chung DH. Targeting Aurora kinase-A downregulates cell proliferation and angiogenesis in neuroblastoma. J Pediatr Surg. (2014) 49:159–65. 10.1016/j.jpedsurg.2013.09.051 24439602 PMC4183462

[120] Keith B Simon MC. Hypoxia-inducible factors, stem cells, and cancer. Cell. (2007) 129:465–72. 10.1016/j.cell.2007.04.019 17482542 PMC3150586

[121] Rotinen M You S Yang J Coetzee SG Reis-Sobreiro M Huang WC. ONECUT2 is a targetable master regulator of lethal prostate cancer that suppresses the androgen axis. Nat Med. (2018) 24:1887–98. 10.1038/s41591-018-0241-1 30478421 PMC6614557

[122] Lu T Wu B Yu Y Zhu W Zhang S Zhang Y. Blockade of ONECUT2 expression in ovarian cancer inhibited tumor cell proliferation, migration, invasion and angiogenesis. Cancer Sci. (2018) 109:2221–34. 10.1111/cas.13633 29737581 PMC6029829

[123] Kosari F Ida CM Aubry MC Yang L Kovtun IV Klein JL. ASCL1 and RET expression defines a clinically relevant subgroup of lung adenocarcinoma characterized by neuroendocrine differentiation. Oncogene. (2014) 33:3776–83. 10.1038/onc.2013.359 24037524 PMC4329973

[124] Rudin CM Drilon A Poirier JT. RET mutations in neuroendocrine tumors: including small-cell lung cancer. J Thor Oncol. (2014) 9:1240–2. 10.1097/JTO.0000000000000301 25122419 PMC4137454

[125] Ban K Feng S Shao L Ittmann M. RET signaling in prostate cancer. Clin Cancer Res. (2017) 23:4885–96. 10.1158/1078-0432.CCR-17-0528 28490466 PMC5609835

[126] VanDeusen H Ramroop JR Morel KL Sheahan AV Sychev Z Lau NA Targeting RET kinase in neuroendocrine prostate cancer. biorxiv [Preprint]. (2019). 10.1101/622415 PMC7415621 32461304

[127] Verrienti A Tallini G Colato C Boichard A Checquolo S Pecce V. RET mutation and increased angiogenesis in medullary thyroid carcinomas. Endocr Relat Cancer. (2016) 23:665–76. 10.1530/ERC-16-0132 27402614

[128] Choi CH Song T Kim TH Choi JK Park JY Yoon A. Meta-analysis of the effects of beta blocker on survival time in cancer patients. J Cancer Res Clin Oncol. (2014) 140:1179–88. 10.1007/s00432-014-1658-7 24671228 PMC11823737

[129] Cole SW Sood AK. Molecular pathways: beta-adrenergic signaling in cancer. Clin Cancer Res. (2012) 18:1201–6. 10.1158/1078-0432.CCR-11-0641 22186256 PMC3294063

[130] Holmes S Griffith EJ Musto G Minuk GY. Antihypertensive medications and survival in patients with cancer: a population-based retrospective cohort study. Cancer Epidemiol. (2013) 37:881–5. 10.1016/j.canep.2013.09.001 24075077

[131] Thaker PH Han LY Kamat AA Arevalo JM Takahashi R Lu C. Chronic stress promotes tumor growth and angiogenesis in a mouse model of ovarian carcinoma. Nat Med. (2006) 12:939–44. 10.1038/nm1447 16862152

[132] Palm D Lang K Niggemann B Drell TLt Masur K Zaenker KS. The norepinephrine-driven metastasis development of PC-3 human prostate cancer cells in BALB/c nude mice is inhibited by beta-blockers. Int J Cancer. (2006) 118:2744–9. 10.1002/ijc.21723 16381019

[133] Hassan S Karpova Y Baiz D Yancey D Pullikuth A Flores A. Behavioral stress accelerates prostate cancer development in mice. J Clin Invest. (2013) 123:874–86. 10.1172/JCI63324 23348742 PMC3561807

[134] Powe DG Entschladen F. Targeted therapies: using beta-blockers to inhibit breast cancer progression. Nat Rev Clin Oncol. (2011) 8:511–2. 10.1038/nrclinonc.2011.123 21808268

[135] Crea F Fornaro L Bocci G Sun L Farrar WL Falcone A. EZH2 inhibition: targeting the crossroad of tumor invasion and angiogenesis. Cancer Metastasis Rev. (2012) 31:753–61. 10.1007/s10555-012-9387-3 22711031

[136] Smits M Mir SE Nilsson RJ van der Stoop PM Niers JM Marquez VE. Down-regulation of miR-101 in endothelial cells promotes blood vessel formation through reduced repression of EZH2. PLoS ONE. (2011) 6:e16282. 10.1371/journal.pone.0016282 21297974 PMC3030563

[137] Fallah J Rini BI. HIF inhibitors: status of current clinical development. Curr Oncol Rep. (2019) 21:6. 10.1007/s11912-019-0752-z 30671662

[138] Harrison MR Hahn NM Pili R Oh WK Hammers H Sweeney C. A phase II study of 2-methoxyestradiol (2ME2) NanoCrystal(R) dispersion (NCD) in patients with taxane-refractory, metastatic castrate-resistant prostate cancer (CRPC). Invest New Drugs. (2011) 29:1465–74. 10.1007/s10637-010-9455-x 20499131 PMC3042040

[139] Heath EI Hillman DW Vaishampayan U Sheng S Sarkar F Harper F. A phase II trial of 17-allylamino-17-demethoxygeldanamycin in patients with hormone-refractory metastatic prostate cancer. Clin Cancer Res. (2008) 14:7940–6. 10.1158/1078-0432.CCR-08-0221 19047126 PMC3085545

[140] Beltran H Oromendia C Danila DC Montgomery B Hoimes C Szmulewitz RZ. A Phase II trial of the aurora kinase A inhibitor alisertib for patients with castration-resistant and neuroendocrine prostate cancer: efficacy and biomarkers. Clin Cancer Res. (2019) 25:43–51. 10.1158/1078-0432.CCR-18-1912 30232224 PMC6320304

[141] Baker LH Rowinsky EK Mendelson D Humerickhouse RA Knight RA Qian J. Randomized, phase II study of the thrombospondin-1-mimetic angiogenesis inhibitor ABT-510 in patients with advanced soft tissue sarcoma. J Clin Oncol. (2008) 26:5583–88. 10.1200/JCO.2008.17.4706 18981463

[142] Gordon MS Mendelson D Carr R Knight RA Humerickhouse RA Iannone M. A phase 1 trial of 2 dose schedules of ABT-510, an antiangiogenic, thrombospondin-1-mimetic peptide, in patients with advanced cancer. Cancer. (2008) 113:3420–9. 10.1002/cncr.23953 18932258

[143] Markovic SN Suman VJ Rao RA Ingle JN Kaur JS Erickson LA. A phase II study of ABT-510 (thrombospondin-1 analog) for the treatment of metastatic melanoma. Am J Clin Oncol. (2007) 30:303–9. 10.1097/01.coc.0000256104.80089.35 17551310

[144] Ebbinghaus S Hussain M Tannir N Gordon M Desai AA Knight RA. Phase 2 study of ABT-510 in patients with previously untreated advanced renal cell carcinoma. Clin Cancer Res. (2007) 13:6689–95. 10.1158/1078-0432.CCR-07-1477 18006769

[145] Yap R Veliceasa D Emmenegger U Kerbel RS McKay LM Henkin J. Metronomic low-dose chemotherapy boosts CD95-dependent antiangiogenic effect of the thrombospondin peptide ABT-510: a complementation antiangiogenic strategy. Clin Cancer Res. (2005) 11:6678–85. 10.1158/1078-0432.CCR-05-0621 16166447

[146] Nabors LB Fiveash JB Markert JM Kekan MS Gillespie GY Huang Z. A phase 1 trial of ABT-510 concurrent with standard chemoradiation for patients with newly diagnosed glioblastoma. Arch Neurol. (2010) 67:313–9. 10.1001/archneurol.2010.16 20212229 PMC6120585

[147] Sahora AI Rusk AW Henkin J McKeegan EM Shi Y Khanna C. Prospective study of thrombospondin-1 mimetic peptides, ABT-510 and ABT-898, in dogs with soft tissue sarcoma. J Vet Intern Med. (2012) 26:1169–76. 10.1111/j.1939-1676.2012.00966.x 22816494

[148] Nakamura DS Edwards AK Virani S Thomas R Tayade C. Thrombospondin-1 mimetic peptide ABT-898 affects neovascularization and survival of human endometriotic lesions in a mouse model. Am J Pathol. (2012) 181:570–82. 10.1016/j.ajpath.2012.05.010 22727957

[149] Campbell N Greenaway J Henkin J Petrik J. ABT-898 induces tumor regression and prolongs survival in a mouse model of epithelial ovarian cancer. Mol Cancer Ther. (2011) 10:1876–85. 10.1158/1535-7163.MCT-11-0402 21844212

[150] Song Z Ren D Xu X Wang Y. Molecular cross-talk of IL-6 in tumors and new progress in combined therapy. Thorac Cancer. (2018) 9:669–75. 10.1111/1759-7714.12633 29603884 PMC5983184

[151] Masjedi A Hashemi V Hojjat-Farsangi M Ghalamfarsa G Azizi G Yousefi M. The significant role of interleukin-6 and its signaling pathway in the immunopathogenesis and treatment of breast cancer. Biomed Pharmacother. (2018) 108:1415–24. 10.1016/j.biopha.2018.09.177 30372844

[152] Rossi JF Lu ZY Jourdan M Klein B. Interleukin-6 as a therapeutic target. Clin Cancer Res. (2015) 21:1248–57. 10.1158/1078-0432.CCR-14-2291 25589616

[153] Melchor L Brioli A Wardell CP Murison A Potter NE Kaiser MF. Single-cell genetic analysis reveals the composition of initiating clones and phylogenetic patterns of branching and parallel evolution in myeloma. Leukemia. (2014) 28:1705–15. 10.1038/leu.2014.13 24480973

[154] Capozzi M VON Arx C DE Divitiis C Ottaiano A Tatangelo F Romano GM. Antiangiogenic therapy in pancreatic neuroendocrine tumors. Anticancer Res. (2016) 36:5025–30. 10.21873/anticanres.11071 27798861

[155] Raymond E Dahan L Raoul JL Bang YJ Borbath I Lombard-Bohas C. Sunitinib malate for the treatment of pancreatic neuroendocrine tumors. N Engl J Med. (2011) 364:501–13. 10.1056/NEJMoa1003825 21306237

[156] Gadgeel SM. Targeted therapy and immune therapy for small cell lung cancer. Curr Treatm Opt Oncol. (2018) 19:53. 10.1007/s11864-018-0568-3 30203184

